# Gut virome and microbiome dynamics before and after SARS-CoV-2 infection in women living with HIV and their infants

**DOI:** 10.1080/19490976.2024.2394248

**Published:** 2024-08-26

**Authors:** Rabia Maqsood, LaRinda A. Holland, Lily I. Wu, Emily R. Begnel, Judith Adhiambo, Prestone Owiti, Bhavna H. Chohan, Soren Gantt, John Kinuthia, Dalton Wamalwa, Ednah Ojee, Barbra A. Richardson, Jennifer Slyker, Dara A. Lehman, Efrem S. Lim

**Affiliations:** a Center for Fundamental and Applied Microbiomics, Biodesign Institute, Arizona State University, Tempe, AZ, USA; b Department of Global Health, University of Washington, Seattle, WA, USA; c Department of Paediatrics and Child Health, University of Nairobi, Nairobi, Kenya; d Center of Virus Research, Kenya Medical Research Institute, Nairobi, Kenya; e Département de Microbiologie, Infectiologie et Immunologie, Université de Montréal, Centre de Recherche du CHU St-Justine, Montreal, Québec, Canada; f Department of Research and Programs, Kenyatta National Hospital, Nairobi, Kenya; g Department of Biostatistics, University of Washington, Seattle, WA, USA; h Department of Epidemiology, University of Washington, Seattle, WA, USA; i Division of Human Biology, Fred Hutchinson Cancer Research Center, Seattle, WA, USA; j School of Life Sciences, Arizona State University, Tempe, AZ, USA

**Keywords:** SARS-CoV-2, HIV, gut, virome, bacterial microbiome, longitudinal, mothers and infants

## Abstract

Microbiome perturbations can have long-term effects on health. The dynamics of the gut microbiome and virome in women living with HIV (WLHIV) and their newborn infants is poorly understood. Here, we performed metagenomic sequencing analyses on longitudinal stool samples including 23 mothers (13 WLHIV, 10 HIV-negative) and 12 infants that experienced SARS-CoV-2 infection with mild disease, as well as 40 mothers (18 WLHIV, 22 HIV-negative) and 60 infants that remained SARS-CoV-2 seronegative throughout the study follow-up. Regardless of HIV or SARS-CoV-2 status, maternal bacterial and viral profiles were distinct from infants. Using linear mixed effects models, we showed that the microbiome alpha diversity trajectory was not significantly different between SARS-CoV-2 seropositive and seronegative women. However, seropositive women’s positive trajectory while uninfected was abruptly reversed after SARS-CoV-2 infection (*p* = 0.015). Gut virome signatures of women were not associated with SARS-CoV-2. Alterations in infant microbiome and virome diversities were generally not impacted by SARS-CoV-2 but were rather driven by development. We did not find statistically significant interactions between HIV and SARS-CoV-2 on the gut microbiome and virome. Overall, our study provides insights into the complex interplay between maternal and infant bacterial microbiome, virome, and the influence of SARS-CoV-2 and HIV status.

## Introduction

Disruptions in healthy gut microbiota can have lasting consequences,^1–4^ and there is a growing evidence that viral infections can lead to significant alterations in both the gut bacterial microbiome and virome.^5–8^ For example, people living with HIV have shown increased risk of inflammation, altered virome and bacterial microbiome, and increased pathogenic viral and bacterial infections, even during antiretroviral treatment (ART).^8–10^ In addition, children who are HIV-exposed but uninfected (HEU) have been reported to have an altered gut microbiome compared to HIV-unexposed and uninfected (HUU) children.^11,12^

SARS-CoV-2 infection has also been observed to alter the gut microbiome for both adults and infants and can cause long lasting microbiota disruption.^7,13–16^ Some studies have found specific gut species to be associated with severity of SARS-CoV-2 symptoms, ranging from mild/asymptomatic to severe. For example, *Faecalibacterium prausnitzii, Bifidobacterium bifidum*, and *Akkermansia muciniphila* were negatively associated with disease severity.^7,14^ Other studies have reported overall lower gut microbiome alpha diversity and richness during versus after SARS-CoV-2 infection, and while the richness and alpha diversity increased after recovery, richness was still lower six months after SARS-CoV-2 infection when compared to uninfected controls.^13,17^ To date, few studies include virome sequence data; however, there is some evidence that SARS-CoV-2 impacts the intestinal virome as well.^18–20^

Given that both HIV and SARS-CoV-2 have been shown to alter gut microbiota, and that more than 39 million people have been living with HIV globally during the SARS-CoV-2 pandemic,^21^ studies are needed to understand whether infection with both viruses has additional effects on the gut microbiota. This could have impactful consequences as gut microbiota are implicated in clinical outcomes such as the risk of persistent diarrhea diseases, one of the leading causes of childhood disability-adjusted life years.^22^ This longitudinal study investigates the impact of SARS-CoV-2 on the gut bacterial microbiome and virome of a mother–infant cohort in Kenya that includes women living with HIV (WLHIV) and women without HIV and their HIV-exposed and unexposed infants. We hypothesized that the gut bacterial microbiome and DNA virome of mothers and infants would be altered due to SARS-CoV-2 infection, both at time of infection as well as post infection. This unique cohort additionally also allowed us to investigate whether maternal HIV infection contributes additional alterations to the gut bacterial microbiome and/or virome in mothers and their infants during and after SARS-CoV-2 infection.

## Methods and materials

### Study population

Women were enrolled in their third trimester (28–42 weeks gestations) at Mathare North Health Center in Kenya, Narobi and were only eligible for cohort participation if they if they were 18–40 years old, planned to breastfeed, and if living with HIV, had received ART for ≥6 months. The health center is in a densely populated, low-income neighborhood and provides maternal and child health, antenatal and HIV care services. Almost all WLHIV received TDF + 3TC+EFV at enrollment. All HIV exposed but uninfected (HEU) infants received nevirapine and zidovudine (NVP+ZDV) prophylaxis from birth until complete cessation of breastfeeding and a negative HIV antibody test (~18 months). Antibiotics were also used by the participants as needed during the study period. Majority of the mothers (70%) and infants (85%) took cotrimoxazole while the rest took amoxicillin (infants 6%, mothers 10%), or other antibiotics (infant 9%, mothers 20%). Infants were immunized according to the standard vaccination schedules (including Bacille Calmette-Guérin vaccine for tuberculosis, polio, pneumococcal vaccine-10, rotavirus, measles, rubella and yellow fever; nearly 100% fully vaccinated).

All infants with maternal consent for SARS-CoV-2 testing with stool (for bacterial microbiome and virome sequencing) and blood samples (for SARS-CoV-2 serology) collected between January 2020 and December 2020 were included; maternal samples were only included if their related infant was included in this analysis. SARS-CoV-2 infection was determined by serology assays detecting antibodies to SARS-CoV-2 nucleocapsid protein, as described previously.^23^ Of 72 eligible infants, 60 were negative and 12 were positive for SARS-CoV-2 in 2020. Eighteen WLHIV and 22 HIV-negative women remained negative for SARS-CoV-2 (controls), while the remaining 13 WLHIV and 10 HIV-negative women tested positive for SARS-CoV-2 in 2020. Participants that had SARS-CoV-2 infection were primarily asymptomatic or mildly symptomatic, with no hospitalizations as previously described.^23^ Of the 95 ever seropositive SARS-CoV-2 samples, there were 54 pre-infection samples and 41 post-infection samples. Stool samples were collected from the study participants during scheduled infant wellness checks at day 4 (month 1), week 6 (month 2), week 10 (month 3), month 6, month 12, month 15, month 18 and month 21. Samples were stored at −80°C and maintained in cold chain during transport from Kenya to the United States.

### Bacterial and viral metagenomic sequencing

Approximately 200 mg of stool was weighed out and diluted to 1:6 with SM Buffer then was vortexed at max speed for three minutes to fully homogenize. Samples were centrifuged at 4° for five minutes at 20,000 g. The centrifuged pellet (metagenomics) was taken through Qiagen’s DNeasy PowerSoil Pro Kit following kit protocol recommendations and then followed by Illumina’s DNA prep for library builds and sequenced on a NextSeq 2000 (2×150). The supernatant (viral) was filtered by a 0.20 µm filter and then processed by a VLP enrichment before extractions. A mastermix of Benzonase (2 µl), Baseline Zero 10× Buffer (100µ), and Baseline Zero DNASE (4 µl) was added to each sample and heated for 37° for one hour. After the heat treatment 1 mL was transferred into bioMérieux’s EMAG for total nucleic acid (TNA) extraction. The TNA was amplified with GenomiPhi V2 (GE Healthcare) before moving into library builds with DNA Prep (Illumina) and sequencing with a NextSeq 2000 (2 × 150). Controls of SM Buffer spiked with lambdavirus DNA were used to assess cross-sample contamination during amplification and sequencing.

### Bacterial microbiome and virome analysis

Illumina sequencing reads (2 × 150) paired end reads, an average of 28,749,393 ± 28,076,646 bacterial metagenomic reads, were quality filtered using BBtools and cutadapt.^24,25^ These reads were then input into krakenuniq to output read counts for assigned bacterial taxa. We then used DBSCAN, a clustering algorithm, on 10% of our samples (*n* = 31) to obtain the cutoff for optimal number of unique k-mer assigned per taxon for false positives and true positives.^26^ For our data, anything below 5683 number of k-mers per taxon was removed. We also used R package decontam, as previously described,^27^ and removed the default called contaminants.^28^ As we only have two features, reads and k-mers, DBSCAN was used as our clustering models, as it is robust at handling outliers and as well as work well with small number of features with large number of samples.^26^

Illumina sequencing reads (2 × 150) paired end reads; an average of 23,552,078 ± 24,293,278 DNA viral metagenomic reads, were quality filtered using BBtools and cutadapt. Quality filtered paired reads for each sample were used to build contigs with metaSPAdes for each sample (DNA stool sample = 303, control *n* = 32).^29^ We built a total of 5,536, 238 DNA contigs from stool samples. We then removed human contigs using bowtie2 (5,415,917 DNA contigs), clustered the remaining contigs via cd-hit (2,621,693 DNA contigs) and then filtered by minimum contig length of 1000 for DNA bp (267,202 DNA contigs).^30,31^ Theses contigs were then input into Cenote-Taker 2, VirSorter 2 and tblastx/blastn (GVD/NCBI NT database) to identify viral contig candidates.^32–35^ After the contigs initially passed each tool, we then used checkV for a final contig quality filtering and provirus screening.^36^ We used contigs with at least medium quality and those which had more viral contigs then host contigs as well as all proviruses identified, as per checkV. This gave us a total of (11,500 viral DNA contigs).

The viral contigs were then used to run blastx against viral RefSeq + Neighboring sequences database (downloaded 2020). Using blastx output, we then used taxonomizr was to assign taxonomy to each contig (family level).^35,37^ The final DNA contigs were then used as databases for which all stool sample QC reads were mapped against to get DNA virome matrix for all subsequent analysis. We used R package decontam, and removed the default called contaminants for DNA viruses and used RPKM (reads per kilobase million) to normalize for contig length.

### Linear mixed effects and PERMANOVA models

We used linear mixed-effects (LME) models (R package nlme, version 3.1–148) to compare changes in richness and alpha diversity across post-partum time (mothers) or month of life (infants) while accounting for repeated measurements (random effects). We used PERMANOVA (vegan, permute and Adonis in R) to assess changes in beta diversity as measured by unweighted and weighted Bray–Curtis distances. Person code (mother or infant), maternal HIV status, post-partum time/month of life (age), time since weaning, antibiotic use, time since SARS-CoV-2 infection were included in the LME and PERMANOVA models to account for exposures and/or possible confounding (fixed effects). For infants, time-since-weaning is an important time variable in addition to age, as introduction to solids has a large impact on the gut microbes in infants.^38,39^ Because infant weaning was collinear with age (Pearson *r* = 0.94, *p* < 0.0001), we included them in models separately as each might have different influence on infant bacterial microbiome and virome. For the models we used two different methods to classify SARS-CoV-2 samples: first, we used SARS-CoV-2 infection status at sample collection time and second, we used if patients were ever versus never SARS-CoV-2 seropositive throughout follow-up in the year 2020. This method allowed us to code samples as controls, pre-SARS-CoV-2 infection, and post-SARS-CoV-2 infection in the models. We also measured time since SARS-CoV-2 infection for women who were ever seropositive to see if there were differences in the gut microbiota over time after infection. We first ran LME and PERMANOVA (Adonis) models with to assess whether outcomes differed between mothers and infants and inform if subsequent analyses should be stratified by person code. After stratifying by person, we included an interaction between maternal HIV status and SARS-CoV-2 status to assess the need for further stratification by HIV status in women.

To assess if there was a change in the richness, alpha and beta diversity of SARS-CoV-2 women in comparison to controls, we modeled an interaction term between SARS-CoV-2 and time postpartum/infant month of life to estimate time point specific effects per SARS-CoV-2 seropositivity status. We also removed the interaction term to re-run the LME and PERMANOVA models to test if for differences in alpha diversity, richness and beta-diversity due to the co-variables (metadata) within the model. For the women who were ever SARS-CoV-2 seropositive, we created interactive models for SARS-CoV-2 infection status at time of collection and time to compare the changes that occur in the alpha diversity and richness before and after seroconversion. We then wanted to analyze if there was a change in the first ever positive SARS-CoV-2 samples from all SARS-CoV-2 negative samples. Only the first positive samples were kept in the models. Linear and PCoA plots were plotted using ggplot2 (version 3.3.1).

### Community states

To obtain the community states present in the stool samples of this study we clustered our weighted beta diversity distance matrix with k-means method for the bacteria. We first used the R package factoextra (version 1.0.7) to determine the optimal number of clusters and then used stats function k-means to cluster the relative abundance at bacterial species level into five groups. To determine associations between community states and time, infant/mother, maternal HIV status, SARS-CoV-2 infection, or antibiotic use, we used R package mclogit (version 0.8.7.2) to perform multinomial logit models with random effects for individual participants; the Benjamini-Hochberg method was used to correct for multiple comparisons. We were unable to cluster the DNA virome data due to high interpersonal beta diversity. We used Kruskal Wallis, while accounting for multiple comparisons, to test for significant differences in the most abundant viral families between mother sand infants.

### Differential analysis

To find differentiating bacteria and viruses, we created models using R package Microbiome Multivariable Association with Linear Models (MaAsLin2) with metadata and controlling for longitudinal samples per patient.^40^ For bacteria and viruses separately, we used all samples to determine taxa that that differentiated mothers and infants. We used the default q-value threshold of 0.25 for significance. We then stratified the samples by mother or infant and assessed other factors, including maternal HIV status, SARS-CoV-2 status, antibiotic use, postpartum time, or time since weaning, that could be associated with specific bacteria in mothers and/or infants. We recalculated the q-value using all results outpupt file and used the updated q-value of 0.05 for significance per factor. Among mothers, there were no specific bacteria significantly associated with SARS-CoV-2 infection, HIV status, antibiotics use or postpartum time. Among infants, differentiating bacteria taxa were significantly associated with age, time since weaning, and SARS-CoV-2 status in infants. Viral contigs were associated with SARS-CoV-2 status at time of collection in infants. No differentiating viral contigs were associated with HIV status, SARS-CoV-2 status or postpartum time for mothers. One contig (*Redondoviridae*) was found to be more abundant in mothers using antibiotics in comparison to mothers with no antibiotics.

### Shared bacterial microbiome and virome

To determine if the infant bacterial microbiome and virome was becoming more similar to the maternal (adult-like) bacterial microbiome and virome, we plotted the weighted Bray–Curtis dissimilarity distances between mothers and infants by age/time postpartum. We then used linear regression to determine if there was a significant change in dissimilarity by maternal HIV status and SARS-CoV-2 infection status.

### Humann3

To find active pathways in the bacterial metagenomic sequences, we ran quality filtered sequences through humann3^41^ using default parameters. This output gave us the abundance of pathways for the samples, which we then used for differentiation. With the pathways’ abundance matrix for the stool samples, we used Maaslin2 on all samples to see differentiating pathways between mother and infant samples. We then subset the data into mother and infants and once again ran Maasline2 to test for additional metadata significance including antibiotic use, infant age/maternal post-partum time, maternal HIV status and time since-weaning. The resulting pathways for significant metadata were plotted as heatmaps using pathway relative abundance.

## Results

### Study population

**Figure 1.**
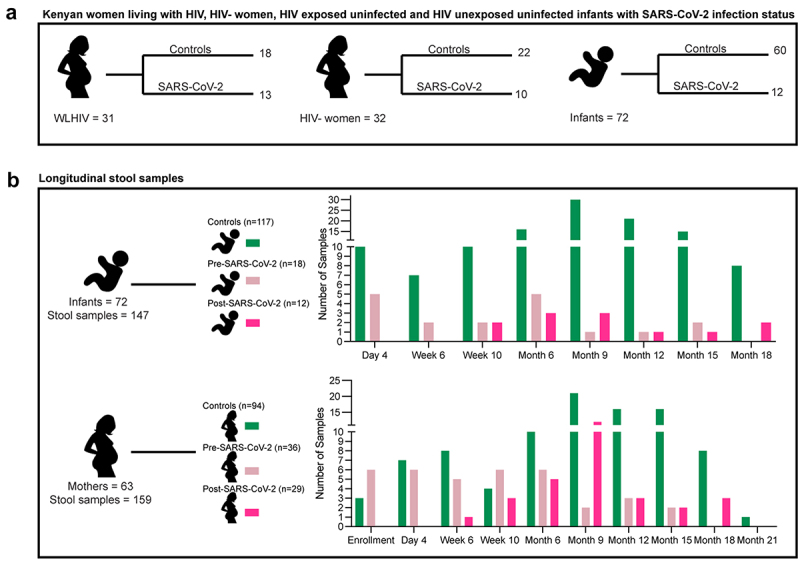
Kenyan women living with HIV (WLHIV), hiv-negative women, and infants.

The Linda Kizazi Study was a prospective birth cohort study of 211 mother-infant pairs in Nairobi, Kenya from 2018 to 2022 (see methods).^23,42^ Between January 1 and December 31, 2020, a subset of 63 mothers and 72 infants consented to SARS-CoV-2 testing and had longitudinal stool samples collected for bacterial microbiome and virome sequencing (Figure 1(a-b)). Thirty-one mothers (women) were living with HIV (WLHIV) and receiving ART and 32 were HIV-negative (Figure 1); 35 infants were HEU and 37 were HUU. SARS-CoV-2 infection timing was determined by serology testing of plasma samples collected approximately quarterly; 23 mothers and 12 infants were positive for SARS-CoV-2 antibodies at any time during study follow-up, which was prior to the availability of SARS-CoV-2 rapid tests or vaccines in Kenya.^23,42^

(a) Number of women and infants stratified by SARS-CoV-2 infection or controls. Women are also stratified by HIV infection. (b) Number of stool samples form infants and all women by timepoint. Colors represent SARS-CoV-2 infection status at time of sample collection.

When stratified by SARS-CoV-2 seropositivity, we found that there was no significant difference in infant age, gestational age at birth, time from weaning, delivery route or maternal CD4 count; however, antibiotic use, maternal age and post-partum time at sample collection were significantly different (Table 1). When stratified by HIV-status, we found no difference by maternal post-partum time, infant age, gestational age at birth, time from weaning or delivery route; however, maternal age at study enrollment was significantly higher among WLHIV (Table 1).

**Table 1.** Population characteristics.

Characteristics	Controls	SARS-CoV-2	HIV-negative	WLHIV	P-value (SARS-CoV-2; HIV status)
Maternal age (median, IQR, range)	27 (6,22)	30 (6.5,18)	26 (8,22)	30 (6,16)	0. 0097; 0.0009
Maternal post-partum month (median, IQR, range)	9 (9,21)	6 (7,18)	9 (6.75, 17)	6 (10, 17)	0.0025;0.28
Infant age in months (median, IQR, range)	9 (6,17)	6 (7,17)	9 (6, 17)	9 (9,17)	0.096; >0.99
Gestational age (median, IQR, range)	38 (0,10)	38 (1.25, 3)	38 (0,3)	38 (1, 10)	0.28; >0.99
Time-since-weaning (median, IQR, range)	6 (9, 16)	3 (6.25, 15)	6 (9, 16)	3 (9, 15)	0.10; 0.81
Antibiotics usage, no. (%)	27 (48%)	29 (51%)	27 (10%)	29 (62%)	0.0004; 0.883
Vaginal delivery no. (%)	94 (91%)	30 (100%)	65 (93%)	59 (94%)	0.21; >0.99
Maternal CD4 count (median, IQR, range)	546 (252.5, 1084)	621 (400, 847)	NA	591 (316.8, 1084)	0.56; NA

### DNA virome and bacterial microbiome differences between mothers and their infants

We performed metagenomic sequencing for bacteria and DNA viruses on 306 stool samples collected longitudinally from the study participants (Figure 1(b)). One maternal sample for the bacterial analysis and three infant samples for the DNA viral analysis were excluded due to low sequencing read depth (<200K reads). An average of 14,147,662 ± 13,612,749 bacterial metagenomic reads per sample and 4,498,593 ± 3,826,239 DNA viral metagenomic reads per sample were analyzed.

**Figure 2.**
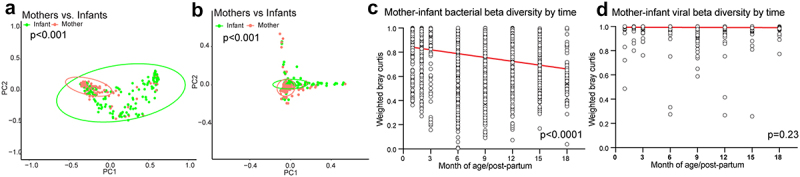
Mother–infant beta diversity over time.

Previous studies have shown that adult and infant gut microbiomes differ substantially,^27,43–46^. Using linear mixed effects models (LME) and PERMANOVA to compare microbiome diversity, we found that maternal and infant samples had significantly different bacterial richness (*p* = 0.0002, Cohen’s d = 0.65(confidence interval 0.30, 1.00)) and alpha diversity (*p* < 0.0001), d = 0.79(0.44, 1.14), but not beta diversity (PERMANOVA, *p* = 0.85). DNA virome richness also differed between mothers and infants (*p* = 0.006), d = 0.61(0.26, 0.96), while virome alpha and beta diversity did not (*p* = 0.13; *p* = 0.62). When comparing all infant and maternal sample Bray–Curtis distances without controlling for time, we found significant differences for bacterial and viral beta diversity (*p* < 0.001, Figure 2(a-b)). Microbiome Bray–Curtis dissimilarity distance between mothers and infants decreased over time regardless of SARS-CoV-2 or maternal HIV status (*p* < 0.0001), indicating infants were converging toward an adult-like microbiome configuration that is resilient to these viral infections (Figure 2(c), Supplementary Figure S1(a-b)). In contrast, the DNA virome beta diversity between mother and infants did not change over time (*p* = 0.23) and maintained high dissimilarity throughout the early life period, regardless of SARS-CoV-2 and maternal HIV status (median maternal-infant dissimilarity = 0.99, Figure 2(d), Supplementary Figure S1(c-d)).

(a) Bacterial weighted PCoA of all samples colored by maternal of infant sample. Statistical significance assessed by PERMANOVA for all infant samples vs. all maternal samples. (b) Viral weighted PCoA of all samples colored by maternal of infant sample. Statistical significance assessed by PERMANOVA for all infant samples vs. all maternal samples. (c) Weighted bacterial Bray–Curtis distance between maternal and infant samples at each time point. Statistical significance assessed by linear regression. (d) Weighted viral Bray-Curtis distance between maternal and infant samples at each time point. Statistical significance assessed by linear regression.

**Figure 3.**
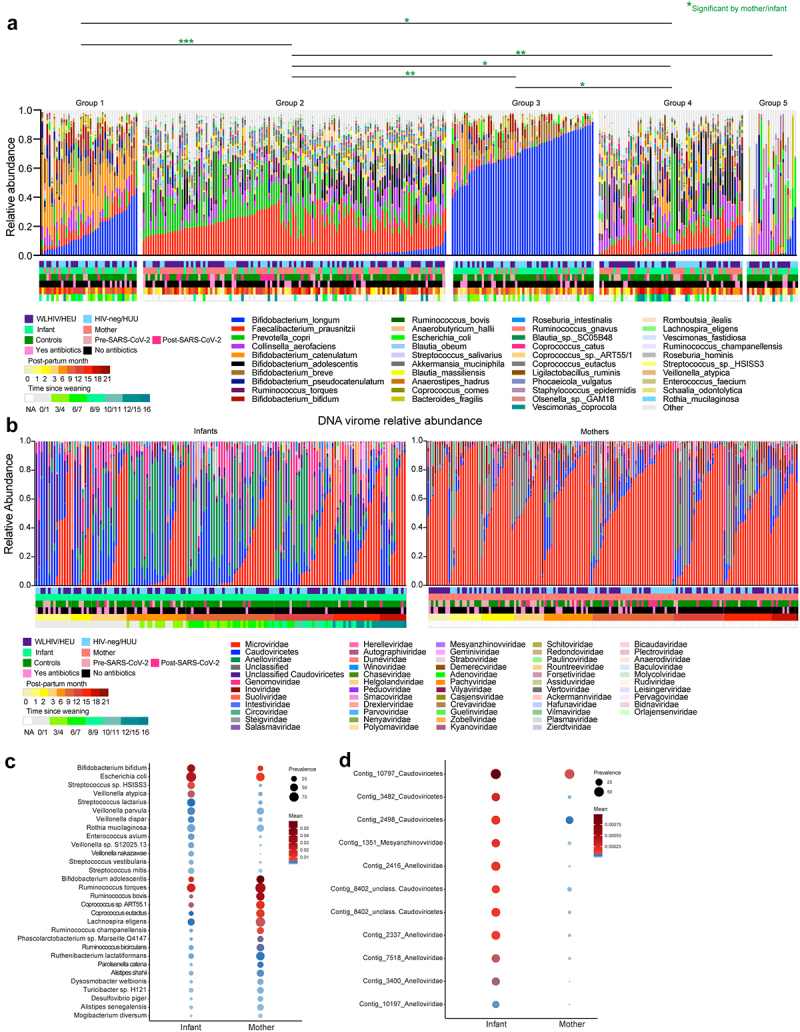
Stool samples community states, relative abundance, and differential analysis of bacterial microbiome and virome. (a) Relative abundance of bacteria at species level, clustered using k-means on weighted Bray–Curtis distances. Plot labeled with community state groups. Statistical significance assessed by multinominal logit models and p-value adjusted for multiple comparison. Significant comparisons represented by color. Multinomial analyses determined if specific bacterial community states were associated with maternal HIV status, mothers versus infants, SARS-CoV-2 status, antibiotic use, postpartum time, or time since weaning. (b) Relative abundance of viruses at family level. Plots are separated by infant or maternal samples and ordered by increasing month of life or post-partum time. (c) MaAslin2Aslin2 assigned differential bacteria for mother and infant samples. (d) MaAslin2Aslin2 assigned differential viral contigs by month of life for infants.

To better understand the community structures, we applied k-means clustering that derived 5 distinct gut bacterial community profiles (Figure 3(a)). Community groups 2 (composed of *Faecalibacterium prausnitzii* (22%) and *Prevotella copri* (15%) abundance) and group 4 (composed of *Faecalibacterium prausnitzii* (12%), *Bifidobacterium adolescentis* (11%), *Collinsella aerofaciens* (10%) abundance) had significantly more maternal samples than infants in comparison to other community groups (Supplementary Figure S1(e-f), p-values between 0.005 and <0.0001). Likewise, 2274 active metabolic pathways were differentiated between mothers and infants (Supplementary Figure S1(g)). Due to high interpersonal variation in the viromes, k-means clustering could not identify distinct virome community clusters. In general, mothers had significantly higher median abundance of *Microviridae, Inoviridae*, and *Suoliviridae* (65%, 1.1% and 0.2%, respectively) than infants (4.2%, 0% and 0.03%, respectively; *p* < 0.0001) (Figure 3(b)). Infants had significantly higher median abundance of *Anelloviridae* and *Genomoviridae* (6% and 2% respectively) compared to mothers (0.01% and 0.4% respectively; *p* < 0.0001).

Multivariable analyses identified 30 bacteria taxa which differentiated mother (e.g., *Bifidobacterium adolescentis* and several *Ruminococccus* species) from infants (e.g., *Bifidobacterium bifidum*, *Escherichia coli*, and several *Streptococcus* and *Veillonella* species) (Figure 3(c)). We also identified 11 viral contigs (mostly *Anelloviridae*) differentially associated with infants (Figure 3(d)). Within just infants, we found several bacterial taxa and viral contigs associated with time and SARS-CoV-2 infection (Supplementary Figure S1(h-k)). *Longibacterium sp. KGMB06250, Faecalibacillus intestinalis* and several *Genomoviridae* contigs were more abundant in SARS-CoV-2 infected infants (Supplementary Figure S1(j-k)). Taken together, these findings indicate that robust microbiome and virome signatures differentiated maternal samples from infants.

### Assessing changes in microbiome and virome trajectory after SARS-CoV-2 infection

Given the dynamic nature of the microbiome, we tested the hypothesis that SARS-CoV-2 infection affected microbiome trajectory over time by LME modeling. Since a subset of women in this study were living with HIV, we assessed if there was an interaction between HIV and SARS-CoV-2. However, because there was no significant interaction between HIV status and SARS-CoV-2 infection on microbiome and virome richness and diversity (Supplementary Figure S1(l)), women were not stratified by HIV status in further analyses. Interaction between HIV exposure and SARS-CoV-2 could not be assessed for infants due to the limited number of HEU infants with SARS-CoV-2 infection (*n* = 7).

**Figure 4.**
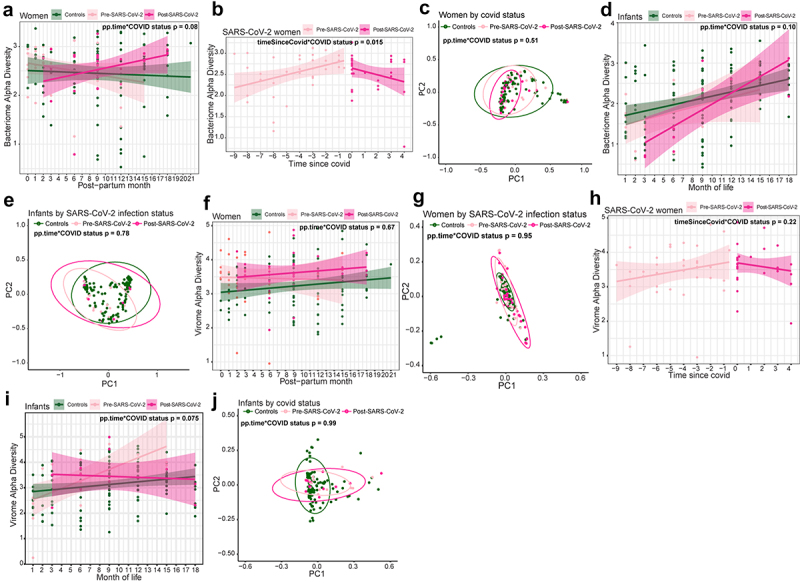
Trajectory change in microbiome and virome after SARS-CoV-2 infection in women and infants. (a) Linear regression plot of maternal bacterial alpha diversities against post-partum time. Colors represent infection status at time of sample collection. Statistical significance of interaction between post-partum time and SARS-CoV-2 assessed by linear mixed effect model. (b) Linear regression plot of SARS-CoV-2 seropositive (same for below) women’s bacterial alpha diversity by time since SARS-CoV-2. Colors represent seropositivity (same for below) status at sample collection. Statistical significance assessed by linear mixed effect model. Statistical significance of interaction between time since SARS-CoV-2 and SARS-CoV-2 positivity assessed by linear mixed effect model. (c) Bacterial weighted Bray-Curtis PCoA of women by SARS-CoV-2 infection status. Colors represent infection status at time of sample collection. Statistical significance assessed by PERMANOVA. (d) Linear regression plot of infant bacterial alpha diversities against month of life. Colors represent infection status at time of sample collection. Statistical significance of interaction between month of life and SARS-CoV-2 assessed by linear mixed effect model. (e) Bacterial weighted Bray-Curtis PCoA of infants by SARS-CoV-2 infection status. Colors represent infection status at time of sample collection. Statistical significance assessed by PERMANOVA. (f) Linear regression plot of maternal viral alpha diversities against post-partum time. Colors represent infection status at time of sample collection. Statistical significance of interaction between post-partum time and SARS-CoV-2 assessed by linear mixed effect model. (g) Viral weighted Bray–Curtis PCoA of women by SARS-CoV-2 infection status. Colors represent infection status at time of sample collection. Statistical significance assessed by PERMANOVA. (h) Linear regression plot of SARS-CoV-2 infected women viral alpha diversity by time since SARS-CoV-2. Colors represent infection status at sample collection. Statistical significance assessed by linear mixed effect model. Statistical significance of interaction between time since SARS-CoV-2 and SARS-CoV-2 positivity assessed by linear mixed effect model. (i) Linear regression plot of infant viral alpha diversities against month of life. Colors represent infection status at time of sample collection. Statistical significance assessed by linear mixed effect model. Statistical significance of interaction between month of life and SARS-CoV-2 assessed by linear mixed effect model. (j) Viral weighted Bray–Curtis PCoA of infants by SARS-CoV-2 infection status. Colors represent infection status at time of sample collection. Statistical significance assessed by PERMANOVA.

We designed interaction LME models to test if the trajectory of microbiome was altered due to SARS-CoV-2 infection over time in women. We found the trajectory changes in bacterial alpha diversity trended differently after infection (post-SARS-CoV-2) compared to SARS-CoV-2 negative women throughout study follow-up (controls), though this did not reach statistical significance (alpha diversity *p* = 0.082, d = 0.37(−0.05, 0.78), Figure 4(a)). We then compared samples prior to infection (pre-SARS-CoV-2) and after infection (post-SARS-CoV-2) by including an interaction term between SARS-CoV-2 infection and time since infection. Alpha diversity increased over post-partum time before SARS-CoV-2 infection, but then markedly reversed to decrease significantly after infection (*p* = 0.015, d = −0.84(−1.50, −0.16), Figure 4(b)). Changes in beta diversity and bacterial richness over time were not associated with SARS-CoV-2 infection (Figure 4(c), Supplementary Figure S2(a)). To assess the significance of individuals variable factors in women, we removed the interaction and found post-partum time and antibiotic use to be significant for changes in beta diversity across all women (post-partum time *p* = 0.020, antibiotic use *p* = 0.030, Supplementary Figure S2(b-c)).

When assessing whether SARS-CoV-2 infection changed infants’ bacterial microbiome trajectory, we found that the richness, alpha diversity and beta diversity of SARS-CoV-2 seropositive infants were not statistically significantly different compared to controls when measuring time by month of life (alpha diversity *p* = 0.10, d = 0.40(−0.08, 0.86); richness *p* = 0.40, d = 0.20(0-.26, 0.67); weighted beta diversity *p* = 0.14; Figure 4(d-e), Supplementary Figure S2(d)), or by time since weaning (richness *p* = 0.63, d = 0.11(−.035, 0.58); alpha diversity *p* = 0.20, d = 0.30(−0.16, 0.77); weighted beta diversity *p* = 0.093). Because changes in the gut microbiome are associated with early life development,^47,48^ we also analyzed infant samples without modeling for SARS-CoV-2 interactions. Bacterial richness, alpha diversity and beta diversity were associated with changes both by month-of-life and time since weaning (richness *p* < 0.0001, d = 1.18(0.67, 1.67); alpha diversity *p* < 0.0001, d = 1.23(0.72, 1.73); beta diversity by month-of-life *p* < 0.001, beta diversity by time-since-weaning *p* < 0.001; Supplementary Figure S2(e)). These findings strongly suggest SARS-CoV-2 infection in microbiome changes over time in adult women but not in infants, potentially due to stronger drivers of microbiome maturation associated with infant development.

Virome alpha diversity, beta diversity and richness over time were generally not associated with SARS-CoV-2 infection in either SARS-CoV-2 seropositive or seronegative women (alpha diversity *p* = 0.67 d = 0.09(−0.32,0.50); beta diversity *p* = 0.95; richness *p* = 0.25, d = 0.24 (−0.17,0.65); Figure 4(f-g), Supplementary Figure S2(f)). However, all women had increasing richness over time postpartum (*p* = 0.017, d = 0.50(0.09, 0.92)), and women that remained seronegative for SARS-CoV-2 had a higher alpha diversity compared to women who were ever seropositive for SARS-CoV-2 (*p* = 0.046, d = 0.53(0.01, 1.04), Supplementary Figure S2f-g). Infant models with interaction between SARS-CoV-2 status and month-of-life showed no significant associations between virome richness (*p* = 0.28,d = −0.26(−0.73, 0.21)), alpha diversity (*p* = 0.075,d = −0.43(−0.91, 0.04)) and beta diversity (*p* = 0.85, Figure 4(i-j), Supplementary Figure S2(h)). Similar to the gut microbiome developmental dynamics, when omitting the interaction from the model, infant virome richness, alpha diversity and beta diversity were significantly associated with changes by month-of-life (richness *p* = 0.010, d = 0.63(0.15, 1.11),; alpha diversity *p* = 0.034, d = 0.52(0.04, 0.99); beta diversity *p* = 0.001, Supplementary Figure S2(i)). These findings are consistent with the dynamic changes in the gut virome during early infant development.^47,49^ Together, these results indicate that the SARS-CoV-2 infection does not substantially alter the gut virome.

### Assessing short term impacts of SARS-CoV-2 infection

**Figure 5.**
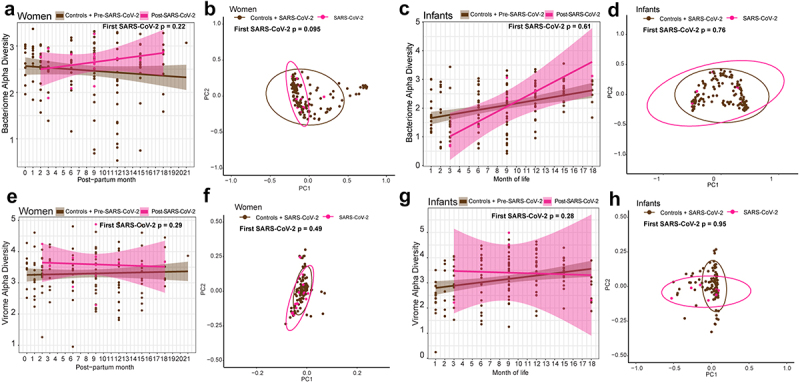
Change in microbiome and virome at first SARS-CoV-2 positive infection visit sample in women and infants against all SARS-CoV-2 negative visit samples.

We next considered whether SARS-CoV-2 might have a more pronounced impact on the microbiome during or immediately after infection. To test this, we compared only the first SARS-CoV-2 seropositive samples to all seronegative samples (including samples prior to seroconversion and all samples from seronegative, uninfected controls). This approach allowed us to identify changes immediately post-infection that might be masked by the overall microbiome stability over time (i.e., prior trajectory analysis) and is more comparable to existing cross-sectional studies. Among women, we did not find significant differences between SARS-CoV-2 seronegative and the first seropositive samples in bacterial richness (*p* = 0.94, d = −0.02(−0.46, 0.43)), alpha diversity (*p* = 0.22, d = 0.28(−0.17, 0.73)), or weighted beta diversity (*p* = 0.095; Figure 5(a-b), Supplementary Figure S3(a)). Findings were similar for infants (richness *p* = 0.93,d = −0.02(−0.50, 0.46); alpha diversity *p* = 0.61, d = 0.13(−0.61, 0.35); month-of-life weighted beta diversity *p* = 0.76, time-since-weaning weighted beta diversity *p* = 0.48, Figure 5(c-d), Supplementary Figure S3(b)). There were also no significant differences between women’s seronegative and first seropositive samples in viral DNA richness (*p* = 0.70, d = 0.09(−0.36, 0.53)), alpha diversity (*p* = 0.29, d = 0.24(−0.20, 0.69)), or weighted beta diversity (*p* = 0.49, Figure 5(e-f), Supplementary Figure S3(c)). Likewise for infants, when modeling for month-of-life, viral richness (*p* = 0.066, d = 0.46(−0.03, 0.96)), alpha diversity (*p* = 0.28, d = 0.27(−0.22, 0.76)), and beta diversity (*p* = 0.95), of their first SARS-CoV-2 positive samples were not significantly different (Figure 5(g-h), Supplementary Figure S3(d)). Thus, there was no evidence that SARS-CoV-2 infection significantly alters the gut microbiome and virome in mothers and infants immediately after infection.

(a) Linear regression plot of maternal bacterial alpha diversities against post-partum time. Colors represent infection status at time of sample collection. Statistical significance assessed by linear mixed effect model. (b) Bacterial weighted Bray–Curtis PCoA of women by SARS-CoV-2 infection status. Colors represent infection status at time of sample collection. Statistical significance assessed by PERMANOVA. (c) Linear regression plot of infant bacterial alpha diversities against month of life. Colors represent infection status at time of sample collection. Statistical significance assessed by linear mixed effect model. (d) Bacterial weighted Bray-Curtis PCoA of infants by SARS-CoV-2 infection status. Colors represent infection status at time of sample collection. Statistical significance assessed by PERMANOVA.(e) Linear regression plot of maternal viral alpha diversities against post-partum time. Colors represent infection status at time of sample collection. Statistical significance assessed by linear mixed effect model. (f) Viral weighted Bray-Curtis PCoA of women by SARS-CoV-2 infection status. Colors represent infection status at time of sample collection. Statistical significance assessed by PERMANOVA. (g) Linear regression plot of infant viral alpha diversities against month of life. Colors represent infection status at time of sample collection. Statistical significance assessed by linear mixed effect model. (h) Viral weighted Bray–Curtis PCoA of infants by SARS-CoV-2 infection status. Colors represent infection status at time of sample collection. Statistical significance assessed by PERMANOVA.

## Discussion

In this study, we investigated how SARS-CoV-2 impacts the gut microbiome of women (both WLHIV and HIV-negative) and their infants. We found that SARS-CoV-2 altered the microbiome; however, the changes we observed were less pronounced than described in several previous studies that found SARS-CoV-2 alters the gut microbiome significantly after infection.^5,7,13,14,16,17,19,20,50–54^ This result could be due, in part, to the clinically mild SARS-CoV-2 cases in our study.^23^ We also found that among women who were seropositive for SARS-CoV-2, bacterial alpha diversity increased before infection, though after infection the alpha diversity showed a trend to be decreasing. This result is consistent with other studies that have similarly showed alteration in the gut bacterial microbiome after SARS-CoV-2 infection.^7,13,14,17^

We also found that women who remained seronegative for SARS-CoV-2 throughout follow-up had a higher DNA virome alpha diversity then women who were ever seropositive. This change in virome diversity due to SARS-CoV-2 has been observed in previous studies, which have also found unique viral signatures associated with SARS-CoV-2.^18–20^ However, unlike these studies, we did not find any viruses that differentiated between women with and without any SARS-CoV-2 infection, which could be influenced by study demographics. Unlike previous studies that mostly included patients from China, our study included Kenyan participants, infants, and women living with HIV. As found in other studies, all women had their gut microbiome diversity influenced by post-partum time.^55^ Another finding consistent with other studies was the association between antibiotic use significant dissimilarity in bacterial beta diversity among women.^56–58^ Additionally, we found that both HIV and SARS-CoV-2 have impact on the gut microbiome as has been found in previous studies.^8,17,18,20,59,60^ Investigating the interaction between SARS-CoV-2 and HIV infection and its impact on gut microbiome and virome is unique to our study, albeit we did not find any significant interactions. However, among SARS-CoV-2 infected women, richness trended to be decreased in WLHIV compared to HIV-negative women (*p* = 0.05; Supplementary Figure S4(a)).

For the infants, we found that time (development) rather than SARS-CoV-2 or other factors we examined, had a stronger effect on the gut microbiome and virome. However, we were able to find several bacteria and viruses that differentiated between infants with and without SARS-CoV-2 infection such as *Longibacterium sp. KGMB06250, Faecalibacillus intestinalis* and several *Genomoviridae* contigs. Previous studies identified specific differentiating bacteria and viruses (*Faecalibaterium prausnitzii, Microviridae* phages) that we did not observe.^7,14,18^ Although not significant, we also found the bacterial alpha diversity in the SARS-CoV-2 seropositive infants trended lower than in the seronegative infants, and the first positive samples trended toward higher viral richness than all seronegative samples (Supplementary Figure S3(d), Supplementary Figure S4(b-c)). Thus, although infant age (i.e., development) was the primary driver of changes in infant gut microbes, SARS-CoV-2 infection also had a potentially subtle impact on the infants’ bacteria and viruses. These findings are comparable to a study of infants ≤2 years old that found bacterial alpha and beta diversity were not significantly influenced by SARS-CoV-2.^50^ Other studies with older children (range 8 days–17 years) showed changes in the bacterial alpha and beta diversity as well as differentiating bacteria.^16,51,52,53^ Since the early life microbiome maturation is a dynamic process that occurs over the first 3–4 years of life to reach an adult-like profile, we expect to see this difference in which the impact of SARS-CoV-2 in older children is more in line with findings in adults.^43,61^

A limitation of the study was the limited number of samples for SARS-CoV-2 positive samples for infants. We were therefore unable to determine the interaction of HIV exposure and SARS-CoV-2 infection in infants. Our results indicate that for children under 2, SARS-CoV-2 has no or limited impact on their gut bacterial and viral microbiomes as compared to drivers of developmental growth. There was also lack of regular swab testing in patients, resulting in us being unable to confirm SARS-CoV-2 re-infections and thus we treated all samples after the initial SARS-CoV-2 seropositivity as pos-SARS-CoV-2 in our analysis. Theses samples were from before SARS-CoV-2 vaccines were available in Kenya, as such we were unable to investigate the effect of IgG responses on the gut microbiome. However, we were able to use this unique longitudinal cohort and conduct comprehensive investigation of the gut bacteria and viruses and the influence of SARS-CoV-2 in WLHIV, HIV negative women and their children. Thus, filling a gap in the existing research literature.

## Supplementary Material

Supplemental Material

## Data Availability

Raw sequencing data have been deposited to the NCBI Sequence Read Archive under accession number PRJNA1045584. Code to reproduce the data analysis presented in this manuscript can be found at: https://github.com/ASU-Lim-Lab/Linda-Kizazi-SARS-CoV-2-and-HIV.git.

## Data deposition

Data are deposited at NCBI Sequence Read Archive under accession number PRJNA1045584.
