# Supplementary material for: Gut virome and microbiome dynamics before and after SARS-CoV-2 infection in women living with HIV and their infants
Source: Gut Microbes. 2024 Aug 26;16(1):2394248. doi: 10.1080/19490976.2024.2394248 (PMC11352790; doi:10.1080/19490976.2024.2394248)
Supplement: Supplemental Material [file KGMI_A_2394248_SM9924.zip › Supplementary figure legends.docx]

**Supplementary figure 1. Analysis of mother and infant differences. (**A) Weighted bacterial Bray-Curtis distance between maternal and infant samples at each time point, stratified by ever SARS-CoV-2 infection. Statistical significance assessed by linear regression. (B) Weighted bacterial Bray-Curtis distance between maternal and infant sample at each time point, stratified by HIV-infection status of maternal samples. Statistical significance assessed by linear regression. (C) Weighted viral Bray-Curtis distance between maternal and infant samples at each time point, stratified by ever SARS-CoV-2 infection. Statistical significance assessed by linear regression. (D) Weighted viral Bray-Curtis distance between maternal and infant sample at each time point, stratified by HIV-infection status of maternal samples. Statistical significance assessed by linear regression. (E) Most abundant bacterial species in each community state group. Colored bacteria rows important for column group. (F) Number of maternal or infant sample by group. (G) MaAslin2 differentiating pathways by mother or infants, clustered using hierarchical clustering. Green square indicates cluster of pathways more abundant in infants. (H) MaAslin2 differentiating bacteria by month of time. (I) MaAslin2 differentiating bacteria by time since weaning. (J) MaAslin2 differentiating bacteria by SARS-CoV-2 infection at time of visit. (K) MaAslin2 differentiating viral contigs by SARS-CoV-2 infection at time of visit. (L) Table for maternal HIV*SARS-CoV-2 status interaction models for bacterial microbiome and virome. Statistical significance assessed by linear mixed effects and PERMANOVA.

**Supplementary figure 2. Change in microbiome and virome after SARS-CoV-2 infection in women and infants.** (A) Linear regression plot of maternal bacterial richness against post-partum time. Colors represent infection status at time of sample collection. Statistical significance assessed by linear mixed effect model. (B) Bacterial weighted Bray-Curtis PCoA of women by post-partum time. Colors represent time of sample collection. Statistical significance assessed by PERMANOVA. (C) Bacterial weighted Bray-Curtis PCoA of women by antibiotics use. Statistical significance assessed by PERMANOVA. (D) Linear regression plot of infant bacterial richness against post-partum time. Colors represent infection status at time of sample collection. Statistical significance assessed by linear mixed effect model. (E) Bacterial weighted Bray-Curtis PCoA of infants by post-partum time. Colors represent time of sample collection. Statistical significance assessed by PERMANOVA. (F) Linear regression plot of maternal viral richness against post-partum time. Colors represent infection status at time of sample collection. Statistical significance assessed by linear mixed effect model. (G) Linear regression plot of maternal viral alpha diversity against post-partum time. Colors represent infection status by SARS-CoV-2 seropositivity. Statistical significance assessed by linear mixed effect model. (H) Linear regression plot of infant viral richness against post-partum time. Colors represent infection status at time of sample collection. Statistical significance assessed by linear mixed effect model. (I) Viral weighted Bray-Curtis PCoA of infants by month of life. Statistical significance assessed by PERMANOVA.

**Supplementary figure 3. Change in microbiome and virome at first SARS-CoV-2 infection visit sample in women and infants against all SARS-CoV-2 negative visit samples.** (A) Linear regression plot of maternal bacterial richness against post-partum time. Colors represent infection status at time of sample collection. Statistical significance assessed by linear mixed effect model. (B) Linear regression plot of infant bacterial richness against month of life. Colors represent infection status at time of sample collection. Statistical significance assessed by linear mixed effect model. (C) Linear regression plot of maternal viral richness against post-partum time. Colors represent infection status at time of sample collection. Statistical significance assessed by linear mixed effect model. (D) Linear regression plot of infant viral richness against post-partum time. Colors represent infection status at time of sample collection. Statistical significance assessed by linear mixed effect model.

**Supplementary figure 4. Supplementary diversity analysis.** (A) Richness of bacteria in women ever positive for SARS-CoV-2 by HIV-infection status. Statistical significance assessed by linear mixed effect model. (B) Linear regression plot of infant bacterial richness against post-partum time. Colors represent infection status by SARS-CoV-2 seropositivity. Statistical significance assessed by linear mixed effect model. (C) Linear regression plot of infant viral richness against time-since-weaning. Colors represent infection status at time of sample collection. Statistical significance assessed by linear mixed effect model.
