# Supplementary figures and images for: Gut virome and microbiome dynamics before and after SARS-CoV-2 infection in women living with HIV and their infants
Source: Gut Microbes. 2024 Aug 26;16(1):2394248. doi: 10.1080/19490976.2024.2394248 (PMC11352790; doi:10.1080/19490976.2024.2394248)

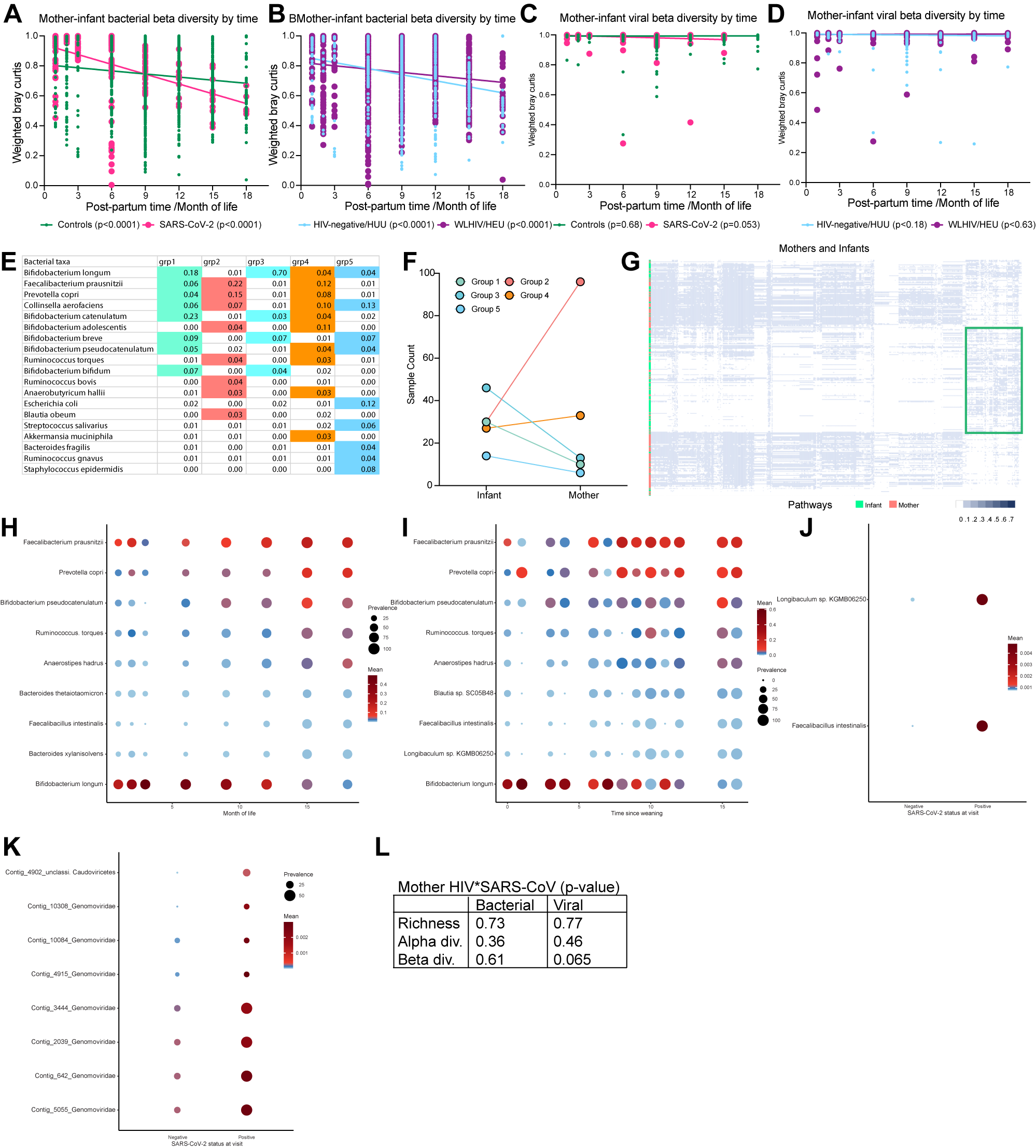

Supplement: Supplemental Material [file KGMI_A_2394248_SM9924.zip › Supplementary Figure 1 (3).tif]

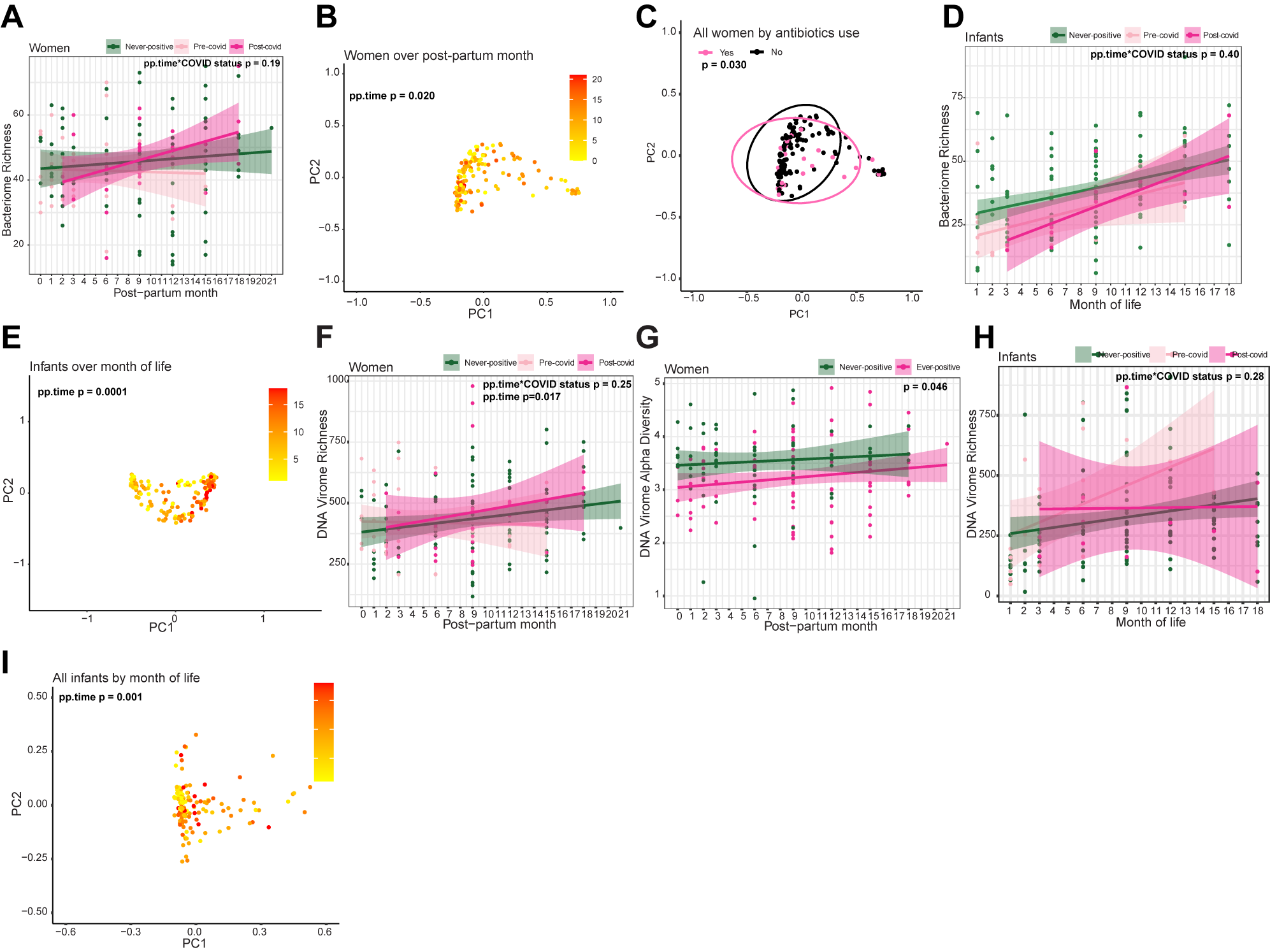

Supplement: Supplemental Material [file KGMI_A_2394248_SM9924.zip › Supplementary Figure 2 (2).tif]

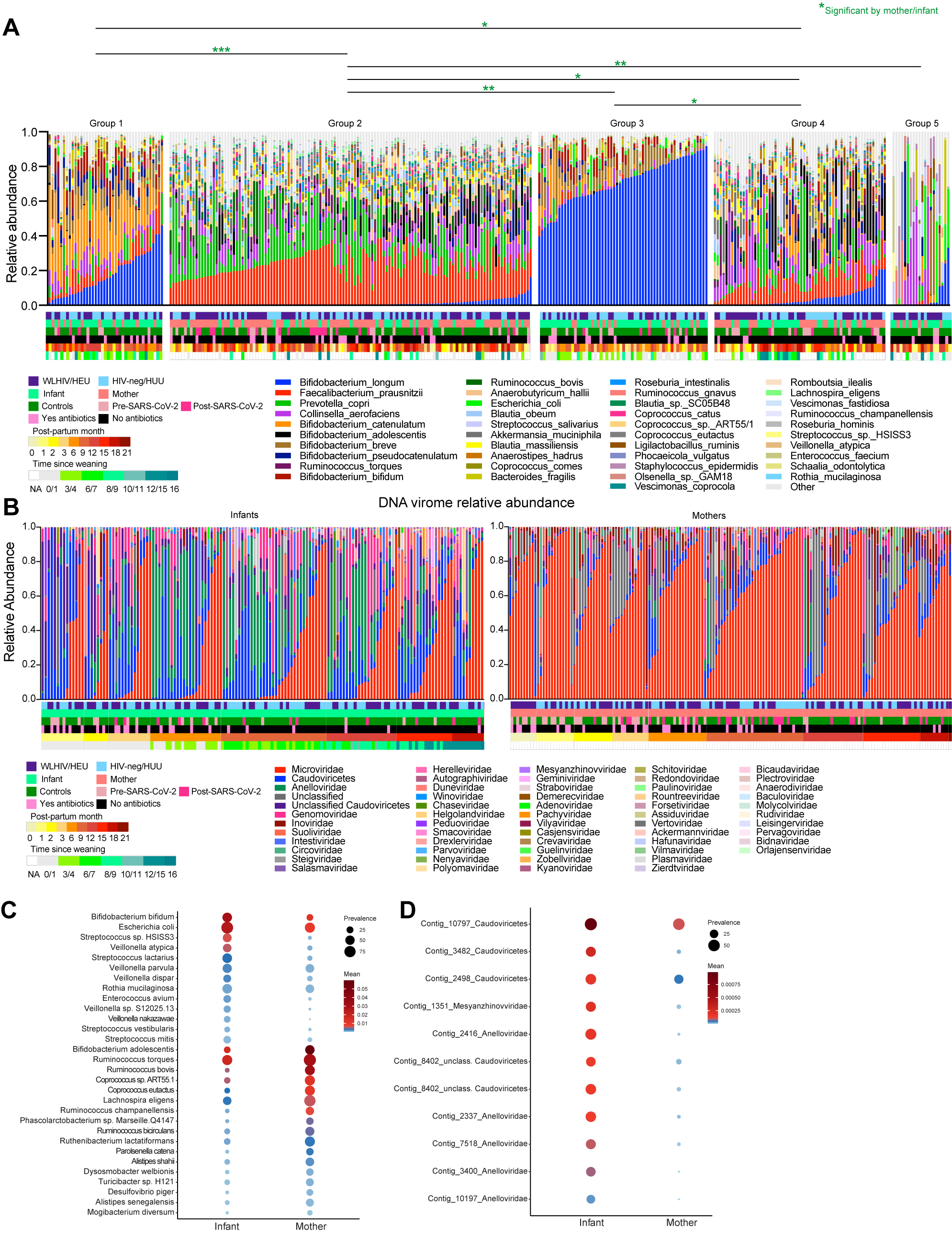

Supplement: Supplemental Material [file KGMI_A_2394248_SM9924.zip › Supplementary Figure 3 (2).tif]

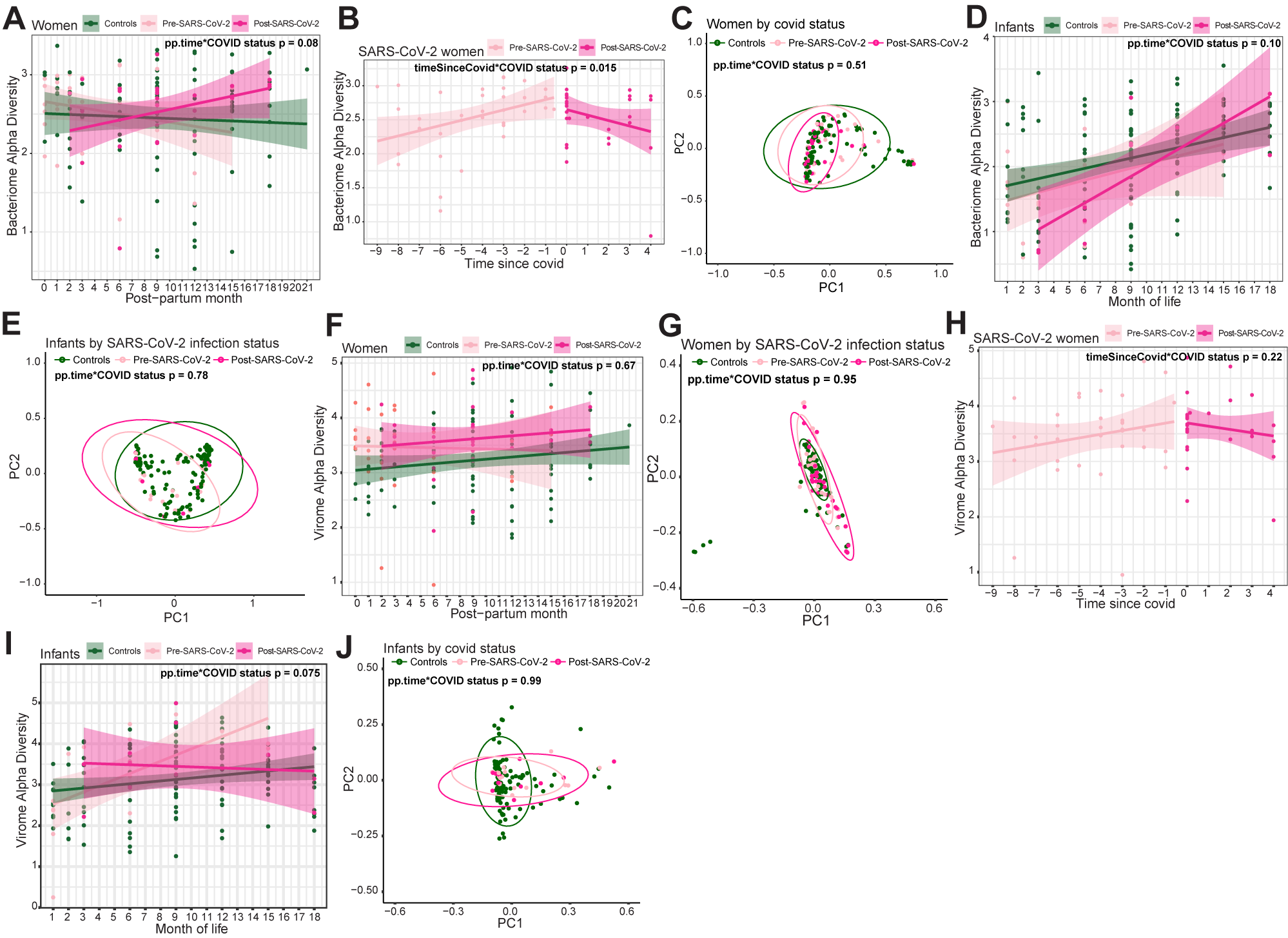

Supplement: Supplemental Material [file KGMI_A_2394248_SM9924.zip › Supplementary Figure 4 (2).tif]
